# Recent Surface Water Extent of Lake Chad from Multispectral Sensors and GRACE

**DOI:** 10.3390/s18072082

**Published:** 2018-06-28

**Authors:** Willibroad Gabila Buma, Sang-Il Lee, Jae Young Seo

**Affiliations:** Department of Civil and Environmental Engineering, Dongguk University, Seoul 04620, Korea; willibroad@naver.com (W.G.B.); dabbi2011@naver.com (J.Y.S.)

**Keywords:** sensors, spatial analysis, remote sensing, Lake Chad, Landsat, surface water mapping

## Abstract

Consistent observations of lakes and reservoirs that comprise the majority of surface freshwater globally are limited, especially in Africa where water bodies are exposed to unfavorable climatic conditions and human interactions. Publicly available satellite imagery has increased the ability to monitor water bodies of various sizes without much financial hassle. Landsat 7 and 8 images were used in this study to estimate area changes around Lake Chad. The Automated Water Extraction Index (AWEI), Normalized Difference Water Index (NDWI), Modified Normalized Difference Water Index (MNDWI) and Normalized Difference Vegetation Index (NDVI) were compared for the remote sensing retrieval process of surface water. Otsu threshold method was used to separate water from non-water features. With an overall accuracy of ~96% and an inter-rater agreement (kappa coefficient) of 0.91, the MNDWI was a better indicator for mapping recent area changes in Lake Chad and was used to estimate the lake’s area changes from 2003–2016. Extracted monthly areas showed an increasing trend and ranged between ~1242 km^2^ and 2231 km^2^ indicating high variability within the 13-year period, 2003–2016. In addition, we combined Landsat measurements with Total Water Storage Anomaly (TWSA) data from the Gravity Recovery and Climate Experiment (GRACE) satellites. This combination is well matched with our estimated surface area trends. This work not only demonstrates the importance of remote sensing in sparsely gauged developing countries, it also suggests the use of freely available high-quality imagery data to address existing lake crisis.

## 1. Introduction

While inland water bodies, which account for 2.5–2.75% of all the water on Earth, constitute a small portion of the Earth’s surface, they are nevertheless essential to sustain a wide variety of aquatic species and some human activities, especially in areas where a dry climate is dominant [1]. For a global carbon cycle, freshwater plays an important role as a sink and a source of the carbon [2]. The legacy use of large-scale inland water bodies spans from the areas of irrigation, energy production, fishery, and transportation to satisfying our domestic needs [3,4]. Harsh climatic conditions threaten the ecological functioning of these water bodies [5,6]. The size and distribution of most freshwater lakes present a challenge for traditional onsite watershed assessment due to time, cost, and logistic constraints. As a solution to this challenge, Landsat imagery and other remotely sensed data sets have been used as effective tools to assess water quantity and quality indicators over water bodies. Mapping inland water body distribution in space and time has proven to be a cost-effective and sustainable method of management [7,8]. A global gridded dataset for the freshwater is a prerequisite for a water resources management and research [9,10,11,12,13]. In Egypt, Ghana, Malawi and Tanzania, this method has been used in the study of inland water bodies, which are inaccessible and plagued with sparse datasets [14,15,16,17]. The applicability of the satellite’s wide areal coverage, re-visits, and multiple freely accessible data channels permits researchers to carry out studies of freshwater bodies in areas where the in-situ observations are sparse. In 2008, the Landsat archive was opened to the public and since then, efforts have been made to atmospherically correct the available images, making distinguishing Earth’s surface features in varying conditions easier [18,19]. For the practical application of remotely sensed images, water body extraction or mapping can be performed using either a hard or soft classification method. The hard classification method comprises of techniques such as the maximum likelihood supervised classification [20], decision tree classifications and minimum distance [21,22,23]. The soft classification is generally a mixed pixel decomposition method [24,25]. This can be achieved by using techniques such as thematic classification [26,27], single band threshold [28,29], the spectral relationship method [30,31] and the water index method, which is the most commonly used method because of its specific application [27,32,33,34]. These methods make use of the reflectivity index of each band and provide water body information based on signature differences between water and other land surface features. Spectral water indices are combinations of surface reflectance at two or more wavelengths that indicate the relative abundance of features of interest. These combinations can enhance water information while constraining other land-cover types as well as eliminating noise components.

Historically, Lake Chad was ranked among the largest lakes in the world. Weather changes, precipitation frequencies and poor irrigation have caused the water level to drop to about 90% of its value in 1963 [35,36,37,38,39]. Following this decrease, extensive information on what might have caused this phenomenon is well documented and available. Coe et al. [36] pointed out that persistent drought was the driving force behind the lake’s continuous decrease while another study tied this tremendous decrease to an increase in irrigation activities around the lake [40]. Studies of the lake’s hydrology were carried out using altimetric measurements of water height to estimate river discharge around the Chari confluence using imperial regression techniques [41]. To compensate for the lack of hydrological data, some researchers reconstructed the past levels and inundated areas within the basin [42]. Annual maximum inundation in the northern pool was observed using Landsat Multispectral; Scanner (MSS) band 7 (0.8–1.1 μm) data from 1973 to 1976 and Meteosat data in the visible channel from 1977 to 1990 [43,44,45,46]. Meteosat thermal maximum composite data was used to account for water covered by aquatic vegetation and the time series of inundated area estimates for Lake Chad from 1986–2001 [47]. Estimates of actual evapotranspiration over the Lake Chad Basin has been thoroughly covered using satellite imagery and GRACE Terrestrial Water Storage (TWS) [48]. An in-depth study of the hydrological variation within the basin using a combination of remotely sensed and hydrological model data-sets to characterize the spatiotemporal variability and multiscale variability of the lake’s hydrological cycle showed that rainfall intensity plays a vital role in lake water availability and identified their bimodal relationship [49]. Using multivariate regression analysis of the time series from 1988–2012 on a sub-regional level, Erik et al. [50] employed two hydrological systems, lake levels and rainfall data, to investigate the correlations and predictive capacities of lake water levels, rainfall and temperature variability to inter-annual variations of harvested maize and millet.

The use of Landsat Thematic Mapper (TM), Enhanced Thematic Mapper Plus (ETM+) and Operational Land Imager (OLI) sensor in delineating the lake’s open water features and providing its area estimates are yet to be covered. Recent surface area estimates are critical to understanding the desiccation story of the basin in recent times [51]. Cadre Harmonisé, a food security analysis tool unique to West Africa, demonstrated that this crisis has contributed to soaring food insecurity, with more than 6 million affected in the region of the four countries surrounding the lake [52]. In the FAO’s response strategy for 2017–2019, aimed at addressing the Lake Chad Basin crisis, part of the strategic framework is to manage the natural resources as well as resource-based conflict reduction by promoting sustainable management of land use, pasture and water resources at the community level [53]. This involves monitoring these resources at cross-border levels. The purpose of this study, therefore, is to determine the area changes of available surface water within the Lake Chad basin from 2003 and 2016 using Landsat TM, 7 ETM+ and 8 OLI imagery. GRACE-derived TWSA changes and some meteorological data from the Lake Chad Basin Commission (hereafter LCBC) were used to complement our area estimates. The resulting series of Landsat satellite data can be used to delineate water features information and to map surface water area changes in the future. In terms of shoreline protection strategies, the information gathered from the extraction of water features using remotely sensed data will be beneficial to management authorities not only in terms of finances but also consistency. Better knowledge of the water coverage duration, beginning and ending dates for the vast range of marshlands surrounding the lake is important for the measurement, modeling and management of marshland ecosystems in this area.

The specific objectives of this study are to analyze the changes in areal surface water coverage in Lake Chad using the appropriate water index derived from Landsat imagery and to investigate the link between hydroclimatic parameters and surface water extent.

We examined the aerial changes in the Lake Chad area over the 13-year period, 2003–2016. A series of 416 Landsat images acquired between 2003 and 2016 were used to investigate these changes and they were compared against GRACE TWSA and rainfall data. We tested four reflectance indices: AWEI, NDWI, MNDWI and NDVI for extraction of the lake surface area from our Landsat data. For consistency reasons, the years 2007 and 2015 were chosen as our test periods. Both years had high quality data. In terms of accuracy, MNDWI gave us the best results and was therefore used to calculate the areal extent during our study period. The changes were then statistically analyzed. This method has been covered extensively with an abundance of techniques which made use of band ratios and individual bands [33,54,55].

## 2. Study Area

**Figure 1:**
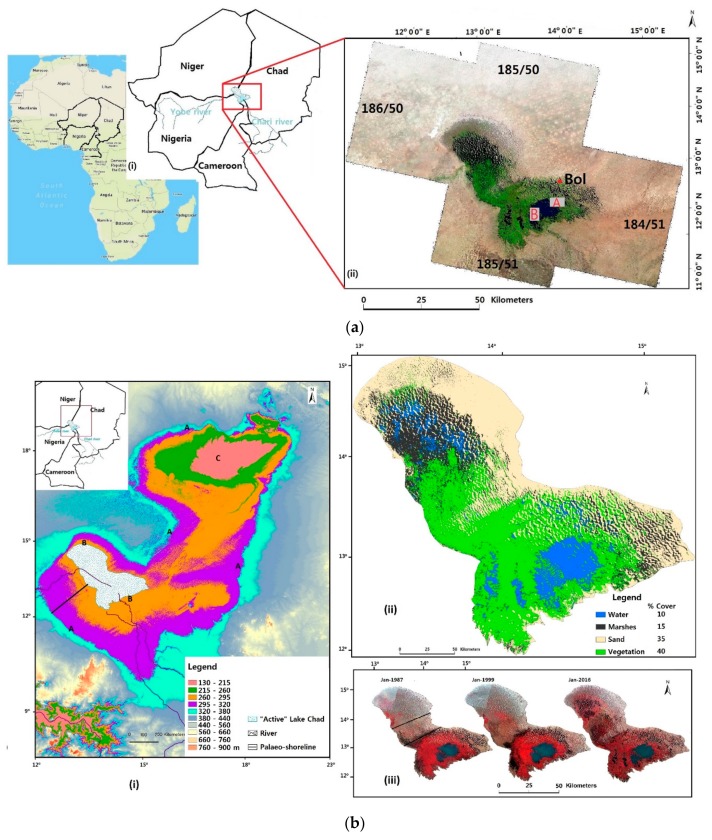
(**a**) (i) Location of Lake Chad Basin with area of permanent water. (ii) Four Landsat images with their respective Path/Row mosaicked to form a complete study site. Point A and B represents the locations of the high-resolution reference data. Bol is the closest village to the lake where most ground hydrological measurements collected by the LCBC. (**b**) (i) SRTM30 DEM of the Lake Chad Basin. A and B represents the shoreline of the “mega” and “active” Lake Chad respectively. The active Lake Chad shoreline has an elevation of about 290 m a.s.l. C is the lowest point within the basin. (ii) Averaged landcover types for July–December 2016 (iii) False color composite image produced using a combination of Landsat TM (4, 3, 2) and OLI (5, 4, 3) bands. The bars in the January-1987 image represent the split described by [38].

The Lake Chad Basin, whose interconnected water system includes Lake Chad (Figure 1a), spans through Cameroon, Niger, Nigeria, Chad and the Central African Republic, providing natural resources for over 30 million people in the region [56]. The basin is situated at an altitude higher than 300 m above the mean sea level where the Sahara Desert meets the Savannah lands, in the middle of the Sahel (Figure 1b(i)). Between 1963 and 2013, Lake Chad, which is a freshwater lake that lies at the center of the basin, has lost about 90% of its water mass, shrinking from ~25,000 km^2^ to 2500 km^2^ [57,58]. This significant loss of water within the basin is evident in the Shuttle Radar Topography Mission (SRTM) Digital Elevation Model (DEM) in Figure 1b(i). The lake shoreline receded from point A to point B. This reduction, which is mainly because of climate change and human factors, threatens the resources and livelihood of the more than 30 million people in the region. Numerous studies have been conducted with the aim of understanding these fluctuations and providing some sort of solution to ameliorate the Lake Chad situation. Lake levels have fluctuated considerably over the past decades [36,37]. Climate variability and environmental degradation were implicated as the main causes of lake level fluctuations. Previous study shows that from 1983 to 1994, water used for irrigation in this area increased fourfold compared to the levels during 1953–1979 [36]. Another study used a hydrological model to simulate the effects of bathymetry, human interactions, and climate variability on the lake level, surface area, and water storage. They found that lake siltation and severe droughts caused the lake to split in 1972. Until the 1990s, the lake failed to merge back into a single lake during the wet seasons. The authors also highlighted irrigation withdrawals as the main cause of water depletion in this area [38]. It is still challenging to evaluate an exact amount of existing open water within the Lake Chad Basin.

There is a crucial need to properly manage whatever amount of open water is left within the basin. Figure 1b(ii) shows that on an average, about 55% of the lake area is covered by vegetation and marshes. Open water occupies about 10% of the lake area. A brief investigation using a false color composite image showed the barrier of land described by Gao et al. [38] was present until the early 80s when vegetation began sprouting over the area (Figure 1b(iii)). From the late 90s to recent times, we can see major changes in terms of vegetation spread. The two horizontal bars on the January-1987 false color composite image represent a large area mainly covered by sand which separated the Northern from the Southern parts of Lake Chad. With clogged irrigation channels and river channels blocked by siltation, water could barely reach the Northern part of Lake Chad. Bdliya et al. [59] analyzed the causes and impacts of invasive species and sedimentation along the Yobe River and found that some of the river channels dried out and as a result, these channels which serve as the lake intake, are filled by sediments. 

Large strips of land colonized by invasive species contributed to the diversion of flow away from the Lake. In the January-1999 false color composite image in Figure 1b(iii), we can still see this split, but this time with some sparse vegetation. In January-2016, the split was covered with more vegetation and marshes. The southern part of the lake, which is mainly fed by the Chari-Logone River, is the most hydrologically active part of the lake. Several rivers in north-eastern Nigeria, including the Yobe River and its tributaries, flow into Lake Chad (Figure 1a(i)).

**Figure 2:**
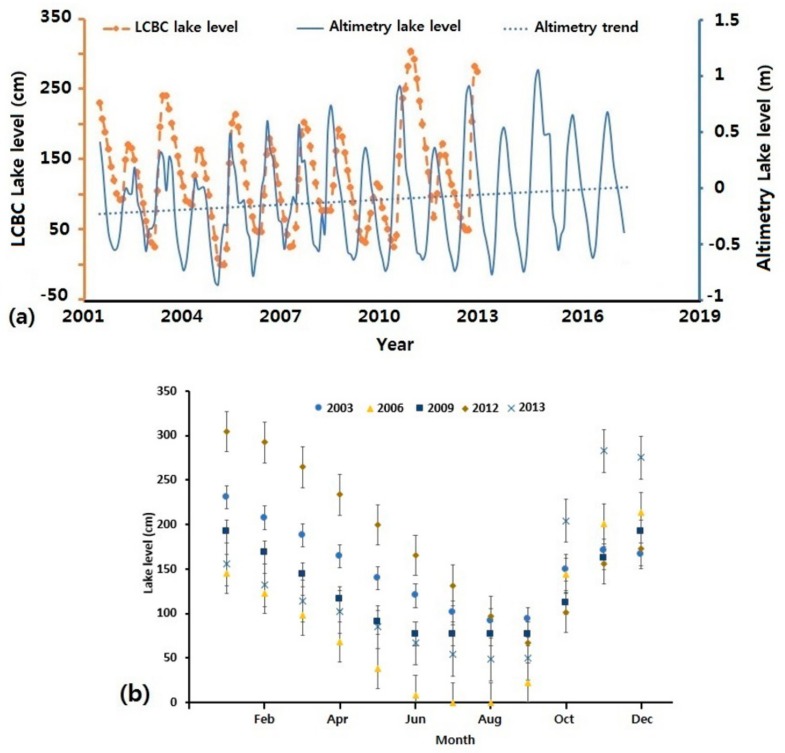
(**a**) Lake levels for 1990–2012. (**b**) Monthly LCBC lake levels at Bol village. The error bars represent the standard deviation for each month.

The climate is dry and hot in the North but mild and humid in the South. During the dry seasons, in the past, the lake’s depth varied from about 2–3 m in the Southern pool, to about 3–6 m in the Northern pool, with an average of about 3.5 m [58]. A recent study showed that during extreme conditions, the mean depth varies between 0.5–2 m in the Southern pool, and from 0–1.8 m in the Northern pool [42]. A fairly linear relationship exists between the water depth and water surface area [58]. As mentioned previously, the lake surface area dropped from ~25,000 km^2^ to its current extent of ~2000 km^2^. In situ lake levels measured from the Bol village station (provided by the LCBC) and altimetry lake levels from Hydroweb can be seen in Figure 2. 

Hydroweb is developed by Laboratoire d’Etudes en Geophysique et Oceanographie Spatiale (Toulouse, France). They use multiple altimetry data to provide water levels for lakes around the world (http://hydroweb.theia-land.fr). We could not obtain lake level data from 2013 onward from the LCBC. As such, altimetry lake levels from Hydroweb were used to see what the trend has been like from that period. Despite the multiple rises and falls as seen in Figure 2a, the lake experienced an upward trend throughout the study period. Inter-and intra-annual variations can be seen during this period, with intra-annual variations reaching ~250 for the in-situ lake levels. Figure 2b presents the monthly in-situ lake levels. In the first half of the year, with little or no precipitation in the lake basin, the average lake level steadily decreases from January-July to about 100 cm. It then increases to about 300 cm from August-November when rainfall increases. In 2009, October, November and December had no record of lake level. A temporal linear interpolation was done between them.

## 3. Materials 

### 3.1. Landsat Imagery

The National Aeronautics and Space Administration (NASA, Washington, DC, USA) launched the first Landsat satellite in 1972 and since then, six satellites have been successfully launched. Generally, as these Landsat satellites orbit the Earth, they record data which are then systematically partitioned into images based on scene location and date. The satellites are equipped with sensors such as the Multispectral Scanner (MSS), which recorded data in four spectral bands and comprised the first group of Landsat missions, Landsat 1–3. Landsat 4–7, which represents the second group, carried either Thematic Mapper (TM) or Enhanced Thematic Mapper (ETM+) sensors, which featured finer spatial resolution and increased radiometric resolution than MSS. The third group, Landsat 8, is equipped with Operational Land Imager (OLI) sensor, which augment the spectral resolution of the TM group with the addition of deep blue and Cirrus bands. This group also has Thermal Infrared (TIRS) sensors on board, which serve as a second thermal band. The U.S Geological Survey (USGS) operates the Landsat mission, which has provided a near continuous record of imagery free of charge since 2009. 

For this study, Landsat (C1 Level-1) ETM+ and OLI/TIRS images were downloaded from EarthExplorer (http://earthexplorer.usgs.gov/), an archive system provided by the USGS (Reston, VA, USA). Four Landsat scenes (Path/Row: 184/51, 185/50, 185/51 and 186/50) were mosaicked to generate the complete coverage of our study area with about 80% of the lake lying within path/row 185/51 (Figure 1a). Data sets were acquired by Landsat Enhanced Thematic Mapper Plus (ETM+, Landsat 7) and Operational Land Images (OLI, Landsat 8) sensors. The images have a resolution of 30 m. This pixel size prevents fine-scale mapping of surface features. However, its 16-day revisit period facilitates studies of the landscape over time [60,61].

**Figure 3:**
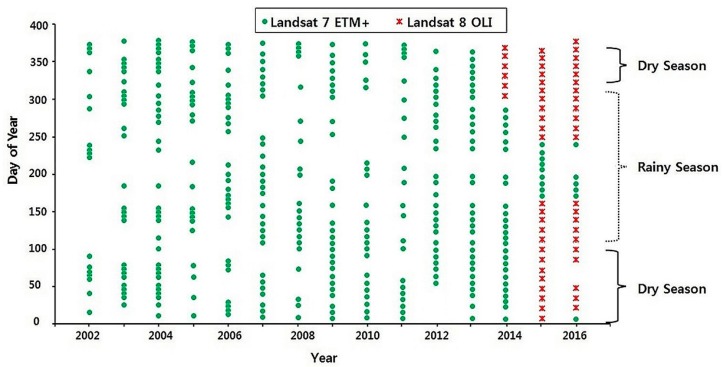
Temporal distribution of Landsat images used in this study.

A temporal distribution of the acquired Landsat images for this study is shown in Figure 3. Figure 3 shows the test years of 2007 and 2015 were also somewhat consistent in terms of data availability throughout the year. Though unevenly distributed in time, acquired Landsat data provided a relatively adequate time series during our study period. The data were pre-georeferenced to UTM zone 33 North projection using WGS-84 datum. Images with less than 5% cloud cover were downloaded for each month for our study area.

As previous studies indicated two seasons over the Lake Chad basin [42,51,58], we focused our studies during the rainy and dry seasons for analyses. A total of 416 images were downloaded between 2003 and 2016. Image processing and lake water extraction and analyses were performed using ENVI 4.1 (Harris Geospatial Solutions, Reston, VA, USA) and ArcGIS 10.4 (Environmental Systems Research Institute, Redlands, CA, USA) software.

### 3.2. GRACE TWSA

For a complete understanding of the hydrological dynamics of the Lake Chad, we used Total Water Storage Anomaly (TWSA) data obtained from the Gravity Recovery and Climate Experiment (GRACE) satellites. The GRACE mission consists of twin satellites following one another at a distance of 220 km in identical Earth orbits [62]. GRACE makes use of the relationship between variations in the gravity field and changes in mass at the Earth’s surface [63,64,65]. In brief, as the twin satellites orbits the earth, the distance between them is used to make gravitational field measurements. Since water availability affects the mass of the earth, changes in water content on the earth surface results to changes in the gravitational field. This affects the movement of the satellites. When the lead satellite passes over a gravity anomaly, its change in speed causes a change in distance with the trailing satellite. This distance is measured using a microwave ranging system. This distance closes as the trailing satellite passes over the same anomaly. Data from this mission is processed by the Geoforschungs Zentrum (GFZ, Potsdam, Germany), the Center for Space Research (CSR) at the University of Texas (Austin, TX, USA) and the Jet Propulsion Laboratory (JPL, Pasadena, CA, USA), before it gets to the final users. TWSA represents a vertical integrated water storage system which comprises of groundwater, surface water, soil moisture, snow water and biological water. When averaged over an area >100 km^2^, it can be used to derive the monthly changes of the total land water storage with an accuracy of about 1.5 cm [66,67,68].

In this study, we used GRACE Level-2 Release 05 solutions (http://grace.jpl.nasa.gov). To reduce the noise caused by orbit resonance during the determination of the Stokes coefficients and aliasing errors, the data set was truncated at 60 degrees and smoothed with a Gaussian filter (300 km) [65]. It is important to restore the signals lost during data processing using an appropriate scaling factor. This must be done before using the data for any analyses. Long et al. [69] demonstrated that scaling factors derived from Land Surface Models showed clear differences over arid and semiarid regions. TWSA products were multiplied by the scaling factors derived from the Community Land Model (CLM) v4.0. It also accounts for irrigation and river diversions, to reduce the uncertainties in restoring the signals from GRACE data.

### 3.3. Rainfall and Lake Level Data

Observed rainfall data were obtained from the Société de Développement du Lac (SODELAC, N’Djamena, Chad), which is a Chadian state enterprise involved in the development of farmland and irrigation projects around the lake [70]. Where the observed rainfall data were lacking, rainfall data from the Tropical Rainfall Measuring Mission (TRMM) was used as a substitute. The rainfall data was extracted from the TRMM3B43 V7 product which is one of the several TRMM rainfall products, containing data from January 2001 to December 2015. This TRMM3B43 dataset has a spatial resolution of 0.25° × 0.25° with a temporal resolution of 3-h. Its spatial extent covers the entirety of the African continent. More information about the TRMM dataset can be found and downloaded freely at https://trmm.gsfc.nasa.gov/.

Lake level data were collected from the Chadian National Meteorological and Hydrological Department (DREM, N’Djamena, Chad). These control centers manage weather stations in some sub-regions around the lake, specifically around the village of Bol. Our study area is characterized mainly by two seasons: a rainy season during which rainfall averages about 300 mm, spanning from May to October, and a dry season from November to March. With the absence of daily rainfall data, which would have allowed for a more precise analysis of the hydrological dynamics around the lake, we used the available monthly data sets. 

## 4. Methods

### 4.1. Pre-Processing

Pre-processing, if properly carried out could account for sensor, solar, atmospheric and topographic effects that affect the quality of Landsat images. Minimizing these image defects by using a specific order of preprocessing methods influences the result analysis [71]. Usually, initial signals from sensors are calibrated to radiance values using gains and offsets that differ among sensors and over time. This is due to sensor degradation. These values are then rescaled to digital numbers as 6-bit or 7-bit (MSS), 8-bit (TM, ETM+), or 12-bit (OLI, TIRS) unsigned integers [72]. In late 2003, there was a permanent failure of the scan-line corrector for the ETM+ sensor aboard Landsat 7. This led to future generated scenes having wedge-shaped gaps and missing pixels (about 22%) within them [73]. To compensate for this, a gap filling method which is based on the local histogram matching technique was applied to the affected images. This method was proposed by the USGS [73,74]. Gap filling was carried out using the *landsat_gapfill.sav* extension toolbox in ENVI., after which, radiometric correction was performed on all the images.

Radiometric correction could be described as the removal of sensor or atmospheric noise, to more accurately discriminate between sensor defects and real ground changes. Values in Landsat images are stored in digital numbers (DN) which represent the electromagnetic radiation captured by their respective satellite sensors. These DNs are scaled representations of radiance and have no physical units [72,75]. Radiometric calibration was carried out by converting the DN to top of atmosphere reflectance for all images using ENVI v5.1 image analysis software. ENVI v5.1 can automatically perform this calibration when supplied with metadata files of each image. Obtained values can be compared to images that have undergone the same level of correction across time or sensors. Cloud contaminated, and hazy images were not included for analyses. As such, atmospheric correction was not performed in this study. It is important to carry out proper pre-processing of downloaded images depending on the depth of the study for which they are used. Failure to perform this task may lead to faulty results and interpretations [27,61,76]. Given the size of our study area, stacking and mosaicking of adjacent images were performed for proper coverage of Lake Chad (Figure 1a(ii)).

### 4.2. Processing

Specific features in each image can be highlighted using spectral indices, transformation, and reduction. Spectral indices manipulation techniques have been designed and tested for highlighting hydrological features [77], vegetation [78], and even fire severity [79], the processing can be achieved to discriminate the aerial changes in the land features. They have widely been used in areas where reference data do not exist to quantitatively examine ongoing changes. The Lake Chad water surface area was extracted individually from each temporal image to detect the surface area changes during our study period. The performance of four different spectral indices was compared for the extraction of surface water (Table 1). Images from Landsat ETM+ 2007 and OLI 2015 were used for this performance analysis. 

McFeeters et al. [54] calculated the NDWI using the reflectance of the green and NIR bands. This was based on the format of the normalized difference of vegetation NDVI developed by Rouse et al. [80], from which numerous water extraction indices have been developed simply by introducing new bands [20,33,81]. The modified normalized water index (MNDWI), which is the most common water extraction index, can constrain plants and impervious areas and reveal subtle water features as well as eliminate shadow effects [33]. The Automated Water Extraction Index is a multiple band index which suppresses classification noise from shadows and other non-water dark surfaces [27]. Amongst the four techniques used for our test periods, the technique with the least amount of error was used to develop the area change estimates of Lake Chad from 2003–2016. 

### 4.3. Performance Evaluation

The water indices in Table 1 are known to have varying performances and depends on the Landsat image conditions and study area size [82]. Each index was calculated from Landsat images during our test period of 2007 and 2015. For proper water extraction, an appropriate threshold is needed for the water and non-water feature segmentation based on each water index image. Previous studies set the threshold values for NDWI and MNDWI to zero [33,54]. However, adjusting these threshold values based on actual situation is necessary. This could achieve a more accurate result for water segmentation. Manually determining threshold value can also be time consuming especially when dealing with many satellite images. As such, to automate this process, some researchers have applied the Otsu’s binarization algorithm for water threshold selection [83,84]. The Otsu method assumes that the image contains two classes of pixels following a bimodal histogram. It then calculates an optimum threshold separating the two classes so that their inter-class variance is maximal [85]. In this study, the Otsu method was used to determine the optimal threshold in distinguishing water and non-water features for each water index image. 

A quantitative assessment of accuracy was conducted to evaluate the performance of NDWI, NDVI, MNDWI, and AWEI indices in extracting surface water estimates over Lake Chad. Since most of the lake is located within the tile (path/row 185/51), we selected our reference data within this tile and outlined its water extent as being the “true” lake extent (Table 2). Reference maps for this study were generated from a free and a commercial image source. For the free reference image source, we made use of Google Earth aerial image. Using visual inspection, an aerial image from Google Earth was used as a visual reference to select an ETM+ image to be used as the reference image. The selected reference image was captured on 8 February 2007. Cloud influence on this image was small. There was no major rainfall or flood event during that month. Its water extent was used as reference to evaluate water area extraction for that period. The commercial image was acquired from Worldview-3 (WV-3). Worldview-3 spectral data have a spatial resolution of 0.31 m. The WV-3 image was provided with 8 bands. The Red, Green, Blue and Near-Infrared bands were used in this study to generate reference maps. The closeness in acquisition dates helped in the reduction of time-dependent effects. 

For the ETM+ sensor image, human visual inspection with the help of google earth aerial image was used in the delineation of the “true” reference map from the selected image. For the OLI sensor image, a multispectral image from Worldview-3 was used in the creation of “true” reference maps. The stratified random sampling method was used to generate 500 points within the boundaries of the high-resolution images for evaluation. We then used pixel-by-pixel comparison method to assess the accuracy of our indices using the reference maps. Finally, an error matrix was created between the results from the water indices and the referenced water bodies. Performance evaluation was performed following recent best practices for remotely sensed data [86,87,88]. Table 3 shows the principle of the error matrix.

In Table 3, a is the number of correct prediction that an instance is negative, b is the number of incorrect predictions that an instance is positive, c is the number of incorrect of prediction that an instance negative, and d is the number of corrections that an instance is positive. 

The accuracy indices include the overall accuracy, kappa coefficient, the product accuracy, and the user accuracy. The Producer accuracy represents the probability that a reference sample is correctly classified. It measures the error of omission. The user accuracy represents the probability that a sample from satellite imagery matches that found in the reference data. It measures the error of commission. The overall accuracy (OA) provides the probability that a randomly selected sample on the imagery is correctly classified. OA can be calculated using Equation (1):

(1) $$\mathrm{OA}=\frac{\mathrm{Number}\;\mathrm{of}\;\mathrm{true}\;\mathrm{positive}+\mathrm{Number}\;\mathrm{of}\;\mathrm{true}\;\mathrm{negative}}{\mathrm{Ground}\;\mathrm{truth}\;\mathrm{pixels}}\times 100$$

The kappa coefficient (κ), which measures the percentage of agreement between the ground truth and segmented water body pixel, was determined using Equation (2):

(2) $$\kappa =\left[\overset{n}{\underset{i=1}{\sum }}P_{ai}-\overset{n}{\underset{i-1}{\sum }}\left(P_{ic}\;x\;P_{ri}\right)\right]\;÷\left[P^{2}-\overset{n}{\underset{i-1}{\sum }}\left(P_{ic}\;x\;P_{ri}\right)\;\right]$$

where *P* = total number of pixels in the reference data, *P_ai_* = total number of correct pixels of the *i*-th category, *P_ic_* = total number of correct pixels of the *i*-th category derived from the classified data, *P_ri_* = total number of correct pixels of the *i*-th category derived from the reference data and *n* is the total number of categories.

Absolute Error (AE) which is simply the difference between the areas detected using the applied method and the reference, was determined using Equation (3):

(3) AE=Referenced area−Estimated area

Summarily, for this study, we first converted the digital number values of the selected Landsat TM, ETM+ and OLI images to at-sensor radiance, followed by a conversion to TOA reflectance. Next, we used the TOA reflectance to form enhanced images of our study area. After applying the equations listed in Table 1 to the processed images, we then defined their specific segmentation thresholds and used them to obtain our water extraction maps. Finally, we used the Overall Accuracy (OA), kappa coefficients (κ) and Absolute Error (AE) to assess the performance of selected surface water extraction methods for Lake Chad.

## 5. Results

### 5.1. Performance Evaluation of AWEI, MNDWI, NDVI and NDWI

**Figure 4:**
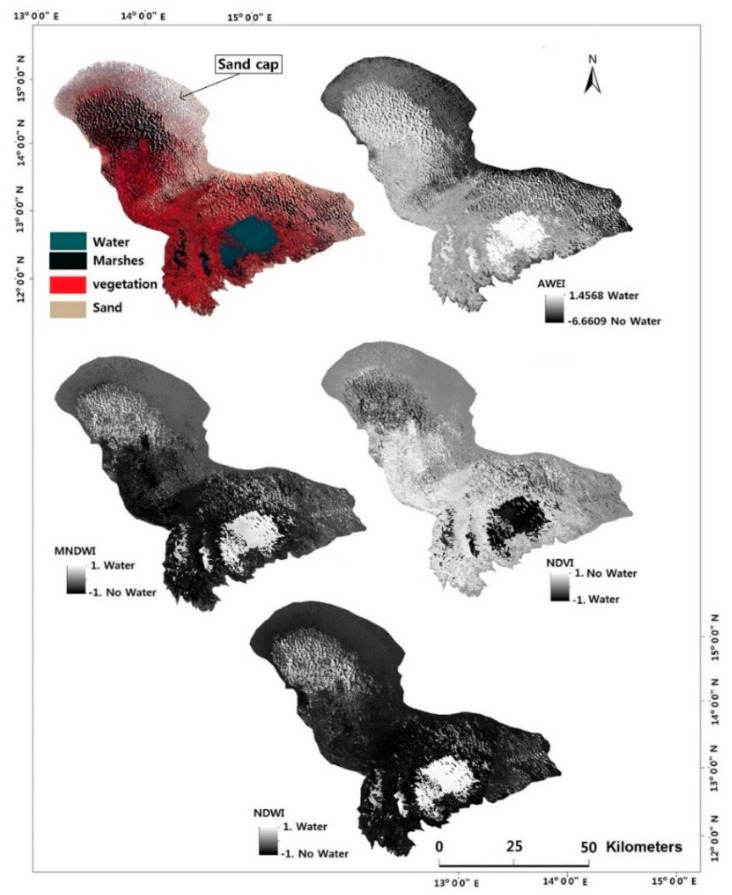
Lake surface water extraction from the different water index for Landsat 8, January-2015 image with a false colour composite (RGB 543) image.

After pre-processing, computed the water index using the equations listed in Table 1. After computation, each image changes to a grey image with high contrast between water and non-water pixels. The results are shown in Figure 4.

The quality of the derived grey scaled index image, as well as the water index in question, are two properties to monitor during threshold segmentation. As seen in Figure 4, the resulting grey scaled index images had a varying degree of separability between water and non-water features. MNDWI, NDVI and NDWI could clearly delineate the patches of water in the northern section of the lake whereas AWEI was poor in this aspect (Figure 4).

This area is largely covered by vegetation, with an open area of sand surrounding most of the northern side of the lake. The southern part of the lake features a permanently open water body surrounded by vegetation and patches of water and sand.

### 5.2. Lake Surface Water Extraction

To evaluate the performance of the indices shown in Table 1, we compared their classification performance using images from Landsat 7 ETM+ and Landsat 8OLI sensor. Though not all indices could obtain a high degree of separability between water and non-water features, the contrast between these features was clear (Figure 4). During threshold segmentation, deviation from the optimal water threshold is a possible cause of faulty results. We used the Otsu method to determine the optimal threshold for distinguishing between water and non-water features for our test period. Table 4 shows the lake surface area estimates of 2007 and 2015. The reference areas of 1350 km^2^ and <2000 km^2^ were obtained from Gao et al. [38], and the Lake Chad Basin Commission, respectively. We see here that the difference in estimated area sizes between 2007 and 2015 Landsat images were not that far apart between the MNDWI, NDVI and NDWI indices. The large difference between the area size of AWEI and the other indices is because AWEI was incapable of delineating much of the surface water around the northern side of the lake (Figure 4). 

#### 5.2.1. Water Extraction Accuracy

The size and geographical location of our study area makes it difficult to obtain accurate ground-truth observational data. This makes validating the accuracy of the lake area estimation methods difficult. High resolution images from other satellite collected around a similar period as the Landsat multispectral image were used for this accuracy assessment. We evaluated the performance of the different methods used to detect lake surface area changes between 2007 and 2015 by calculating their respective absolute errors, overall accuracy and kappa coefficient. A reference aerial image from Google Earth was used to assess the accuracy of the lake area estimate result from the Landsat ETM+ data. For the Landsat OLI data, a reference data from Worldview-03 was used to assess the accuracy of the lake area estimate result (Table 2). 

**Figure 5:**
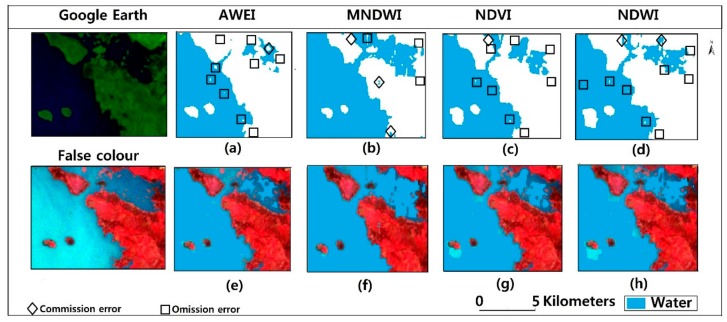
Performance evaluation of AWEI, MNDWI, NDVI and NDWI. (**a**–**d**) are binary images from AWEI, MNDWI, NDVI and NDWI respectively highlighting possible errors. (**e**–**h**) are water pixels overlaid on false composite of the reference image.

Pixel-by-pixel comparison was used to generate error matrixes. These error matrices were used to obtain the overall accuracy and kappa coefficient. The Google Earth aerial image in Figure 5 shows an open water and vegetated land. As an example, we see in Figure 5 that AWEI and NDVI did not perform well in delineating water from its surrounding features as compared to MNDWI and NDWI. Some water pixels were misclassified as non-water features.

In contrast, MNDWI and NDWI performed well in extracting water pixels in this area. MNDWI, NDVI and NDWI have an overall accuracy greater than 90%. MNDWI and NDWI had kappa coefficients greater than 0.9. AWEI had an overall accuracy of 86% with the smallest kappa coefficient of 0.82. According to Table 5, the accuracy is relatively stable among the techniques, and all the water indices can achieve water extraction with an overall accuracy of greater than 80%. The kappa coefficient from the four indices is in the range of 0.8–0.9. For 2007, AWEI had the lowest kappa coefficient of 0.82. MNDWI and NDWI performed well with an averaged overall accuracy of 94.45% and a kappa coefficient of 0.89 between them.

**Figure 6:**
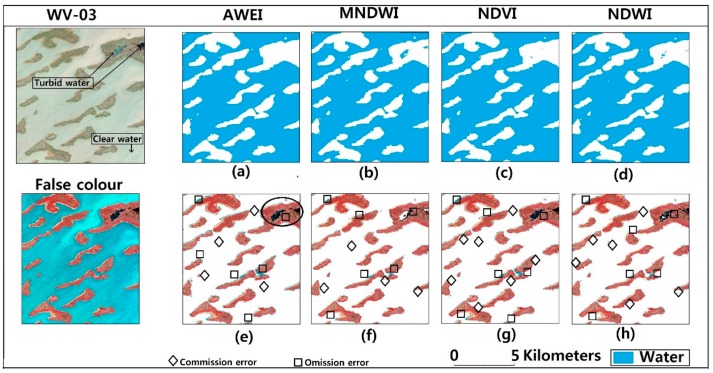
Comparison of the performance of AWEI, MNDWI, NDVI and NDWI on water extraction for the OLI image. (**a**–**d**) are binary images from AWEI, MNDWI, NDVI and NDWI respectively. (**e**–**h**) are water pixels overlaid on false composite image to highlight possible errors.

The performance of AWEI, MNDWI, NDVI and NDWI obtained from our OLI image was assessed using a high-resolution image from Worldview-3 (Table 2). An area with clear and turbid water, vegetation and sandy edges was selected for this analysis (Figure 6). Looking at the first row of Figure 6, we see that the four processes did well in delineating clear water features. The producer and user accuracy are greater than 90% for all the indices. However, only MNDWI could barely delineate turbid water around the chosen area. MNDWI had an overall accuracy of 97% and a kappa coefficient of 0.91 which were both highest amongst the others (Table 5).

Based on accuracy, MNDWI for our test years had an overall accuracy of about 96.35% and a kappa coefficient of about 0.9. This was the highest when compared with the indices used in this study. MNDWI was therefore used to calculate the changes in the Lake Chad area during the period of 2003–2016 using multi-temporal Landsat images.

#### 5.2.2. Optimal Threshold for MNDWI

**Figure 7:**
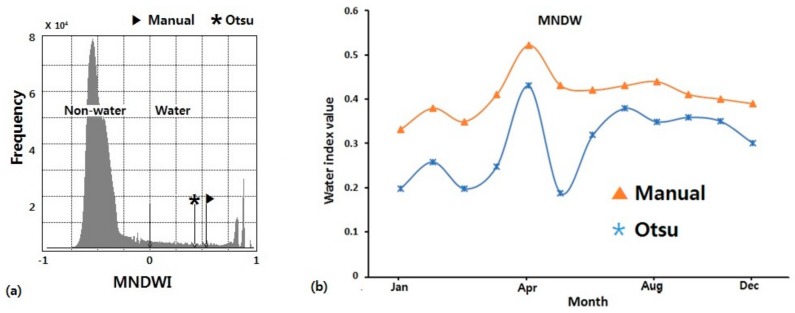
(**a**) Histogram of MNDWI image showing specific locations of water thresholds for L8 12-January 2015. (**b**) Comparison between MNDWI thresholds derived from the Otsu and Manual threshold selection methods. Landsat 8 image acquisition dates 12-January, 13-February, 17-March, 17-April, 20-May, 21-June, 7-July, 25-September, 27-October, 28-November and 30-December 2015. (Landsat 7 image acquisition date: 21-August-2011).

Any selected thresholding method should not affect the ability of the water indices in delineating water and non-water features. To be quite certain about our threshold segmentation method, we evaluated its dependency to MNDWI. This was done by carrying out a brief comparison between the thresholds obtained using the Otsu algorithm and those obtained manually for the year 2015. Manual thresholding was carried out mainly through on-screen visualization with the help of the histogram obtained from our grey scale MNDWI images for that year. Figure 7a shows a contrast between the manual threshold, a conventional 0 threshold and the Otsu’s threshold. From the analysis of 12 images, the deviation between the manual and Otsu thresholds is small (Figure 7b). 

**Figure 8:**
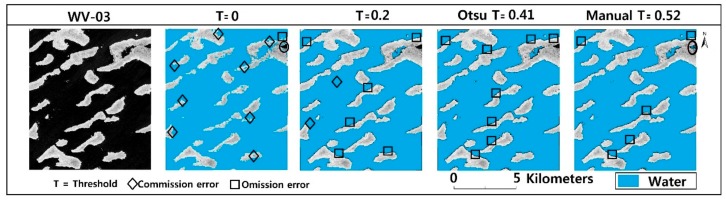
Water maps derived from different thresholds. Landsat date 12 December 2015. The circle represents an area of turbid water.

There was a high accuracy recorded between the selected thresholds for this analysis. The threshold of 0 had an overall accuracy of 80% which was the lowest (Table 6). This was due to numerous commission errors, as seen in Figure 8.

The Otsu and the manual threshold values had an overall accuracy of 95.8% and 98.6% respectively. The accuracy of deviation of derived water maps by the Otsu and manual thresholds for MNDWI was less than 3%. From this analysis, we see that the chosen water extraction method for this study is dependent on our threshold selection method. We can obtain high accuracy result by applying the Otsu thresholding method. Based on this analysis, the thresholds for delineating water and non-water features for MNDWI were determined using the Otsu algorithm. 

**Figure 9:**
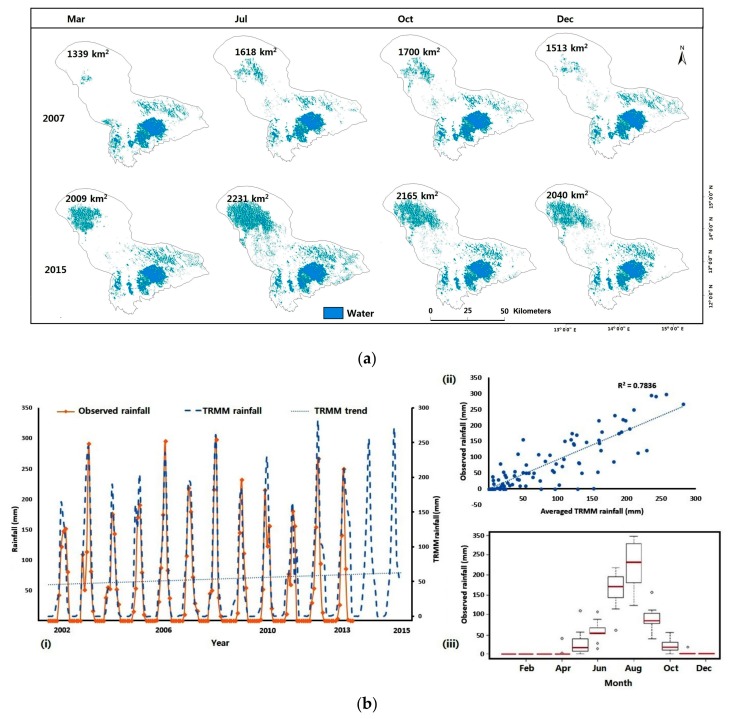
(**a**) Estimated lake surface water area changes for our test years. These years were chosen as representative examples of the 13-year period. (**b**) (i) Monthly rainfall estimates from the LCBC and TRMM 3B42; (ii) Scatter plot comparing their monthly mean; (iii) Boxplot of the observed rainfall estimates.

Figure 9a shows estimated lake surface area maps for March, July, October and December for our test years 2007 and 2015 placed side by side. The calculated area consisted only of water pixels. For the selected months, area estimates revealed a significant increase in lake water extent for 2015 when compared to 2007.

We also see in Figure 9a that the water decrease mostly affects the Northern part of the lake, visually confirming that the lake recedes from North to South. Judging from the trendline, rainfall observations showed a slight increase during our study period (Figure 9b(i)). This region is primarily affected by two seasons: the rainy and dry. During the rainy season which spans from May to October and peaks between July and August, average rainfall is usually greater than 200 mm. This area also recorded occasional rain in April. Rainfall in July, August and September have the highest variations between the years. On the other hand, the dry season, which starts from November, has no rainfall between the months of January and February. This bimodal relationship is seen in Figure 9b(iii), which presents a box plot of monthly rainfall measurements from the LCBC.

### 5.3. GRACE-TWSA: An Integrative Indicator of Lake Chad Hydrological Dynamics

GRACE satellites data processing is continually evolving. Their gravitational observations have been identified as a valuable proxy of water availability over large areas, with consistent validated data free from cloud effects [89,90]. GRACE allows for measurements of the Earth’s gravity field, producing measurements with greater magnitudes. These measurements can be used in monitoring changes in water and ice around the globe, ocean surface and deep currents, as well as provides vital information in the measurement of surface and groundwater changes [91,92,93]. Changes in the TWSA over the lake area has a direct influence on the hydrological and the ecological dynamics around the lake [49]. TWSA was plotted as a representative metric to help illustrate the pattern of the Lake Chad’s area changes during our study period. This was carried out simply to investigate if the estimated area was in line with the TWSA estimates. 

**Figure 10:**
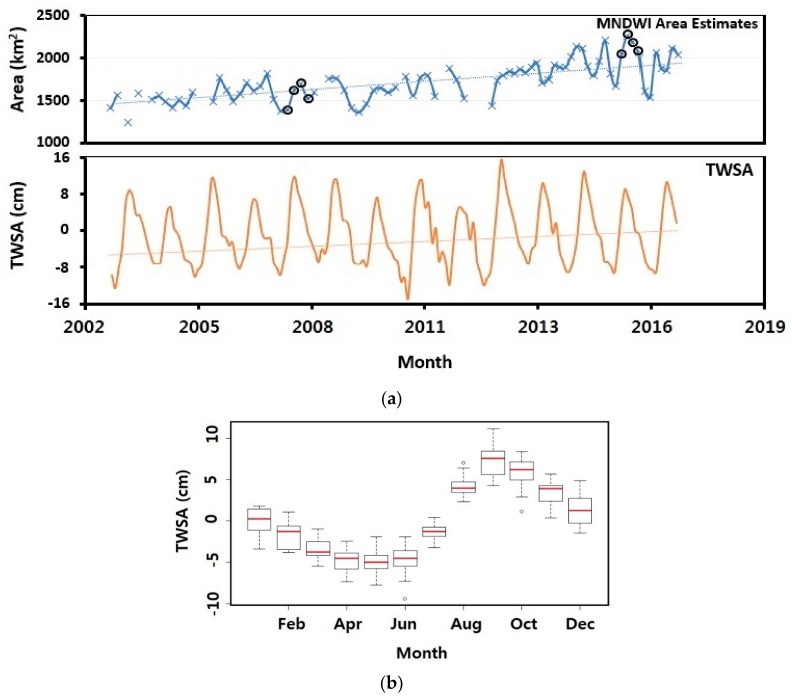
(**a**) Averaged monthly area and averaged monthly TWSA averaged over Lake Chad. The orange line represents the linear trend of the TWSA. The black circles represent selected monthly areas estimates of 2007 and 2015 illustrated in Figure 9a. (**b**) Monthly TWSA boxplot.

The estimated lake area experienced an increase in seasonal fluctuating tendencies. The R-squared value is 0.47 which is indicative of a high variability. Besides the seasonal fluctuations, the difference in area between 2003 and 2012 are small as compared to those between 2013 and 2016 during which the lake area estimates peaked into the 2000 km^2^ range with greater fluctuations (Figure 10a). The TWSA trend over the Lake Chad basin slightly supports the variation in the estimated lake area. The most significant decline in the TWSA occurred in late 2008 followed by its continuous increase.

## 6. Discussion

Lake Chad plays an important role in supporting urban development and serving millions of people across the countries it borders. Therefore, proper management of this water resource is necessary. Given its size and location, monitoring its area using freely available remote-sensing observations will help in proper management of the lake at a lesser cost.

A recent record of the Lake Chad’s area dynamics (2003–2016) was investigated using high spatial resolution (30 m) Landsat data. Due to cloud cover, some downloaded data set collected mostly during the rainy season was disregarded for this study (Figure 3). Insufficient data may lead to estimated area uncertainties. However, we believe the observation frequency of at least two years covering the dry and raining season could help in this study. As such 2007 and 2015 were chosen based on cloud free data and reference data availability. AWEI, MNDWI, NDVI and NDWI indices were used to extract the lake area during this period. The performance of these water index can vary depending on the water type, atmospheric and land surface conditions [27]. The results of the four indices show that the overall accuracy of water features delineation was greater than 80% and the overall kappa coefficient, greater than 0.8. In terms of accuracy, MNDWI stood out with an averaged overall accuracy of 96.3% and kappa coefficient of 0.9. MNDWI was therefore used to delineate water from non-water features for our study area. MNDWI overcomes the shortcomings of its predecessor, the NDWI by using Shortwave Infrared band to replace the Near Infrared band used in NDWI. The MNDWI maximizes the difference in spectral features. It enhances water features by restraining the non-water features within a Landsat image. Here, we presented our results along with: (1) Threshold analysis, (2) Aerial changes in the Lake Chad, and (3) Error analysis.

### 6.1. Threshold Analysis

The potentials of a suitable threshold method to delineate water from non-water features with high accuracy over time was tested for this study. Thresholding significantly affects the efficiency of mapping water features and it is highly influenced by the judgement of the user [94]. With the availability of high quality reference data for accuracy assessment, an optimal repeatable threshold method can be established and verified. Water features within the lake were delineated from non-water features using the Otsu method. The generality of the Otsu method was tested by comparing its outputs to those obtained manually with the help of the gray image histogram for the year 2015. In doing so, we also tested the performance of the widely used 0-threshold and a 0.2-threshold in our study area (Figure 7a).

A threshold of 0 will normally allow for rapid water feature delineation from other components. However, the classification accuracy at subpixel level based from Worldview-3 high resolution image showed that the Otsu and Manual threshold performed better than the 0 threshold in this area (Figure 8). The threshold of 0 misclassified a significant amount of non-water features as water features (Figure 8). It had an overall accuracy of 80% and kappa coefficient of 0.78. Lowest amongst all the threshold compared (Table 6). The Otsu’s threshold of 0.41 and the manual threshold of 0.52 had an overall accuracy of 96.8% and 98.6% and a kappa coefficient of 0.93 and 0.96 respectively. Judging from Figure 7b, there is a huge leap from a threshold of 0 to 0.41. We decided to test a threshold of 0.2 which lies between them to see if it had any significant changes in delineating water features. The 0.2 threshold had an accuracy of 96% and kappa coefficient of 0.91. The difference in accuracy between the manual threshold and all those used in this study was in its ability to identify some turbid water pixels as seen in Figure 8. However, when using MNDWI for delineating water features for our study area, a threshold value of 0.2–0.5 will get a high degree a separability in delineating water from non-water features. The root mean square deviation between the Otsu and manual threshold was ~0.1. These experimental results show that the Otsu algorithm could be effectively used in place of manual threshold for feature segmentation of our study area.

### 6.2. Estimated Area and Surface Changes

Lake area estimates for our study area were obtained by applying the optimal threshold to MNDWI. As Figure 8 shows, MNDWI was highly accurate in delineating clear water in Lake Chad and achieved an extraction accuracy greater than 95%. However, we encountered some difficulties in extracting few turbid water pixels after applying the Otsu method of segmentation. Most of these turbid water pixels were misclassified (Figure 6 and Figure 8). Nonetheless, this occupied less than 5% of the extracted area of the affected images. 

For this study, a total of 78 monthly area covering the entire lake was generated. Generally, there is a slight uptrend in estimated lake area during our study period. The slight increase in lake area is in line with a study which showed improving wet conditions in the last two decades with 2002–2014 described as the wettest period. The authors attributed this to increasing rainfall in the area and the lack of any major long-term drought besides the usual seasonal fluctuations [51]. Lake Chad’s average area from 2003–2016 was estimated to be 1694 km^2^ with a standard deviation of about 233. The largest areas of 2087, 2182 and 2231 km^2^ were recorded in October 2013, September 2014 and July 2015 respectively. This corresponds to the rainy season. The smallest areas of 1242, 1325 and 1379 km^2^ were recorded in March 2003, December 2006 and February 2009 respectively. This period corresponds to the dry season. From Figure 10a, we see that the lake area has a phase from 2003–2012 where the area changes fluctuated within the 1000 km^2^ range. The average area from 2003–2012 was about 1563 km^2^. From 2013–2016, area estimates entered the 2000 km^2^ range with higher seasonal fluctuations. An averaged area of about 1876 km^2^ was recorded for that period. Increase in lake area could be because of no major ongoing irrigation scheme going on in the Northern section of the lake as reported by the LCBC [95]. Increase in estimated lake area could also be partially explained by the slight increase in altimetric lake levels (Figure 2a). Between 2008 and 2014, there was a yearly increase of about 0.4 m/year in altimetry lake levels [51]. Lake area estimates also match with an increasing agricultural productivity. This was reported by a recent study where the authors revealed that locals had experienced an increase in maize yields from 2010 upward compared to a mild harvest during the early 2000s [50]. A possible reason for such an increase in agricultural productivity is more surface water. An increasing trend in GRACE TWSA around this area also backs the slight increase in lake area (Figure 10a). Summarily, increased rainfall, lake levels and a recent halt in irrigation schemes on the lake are all consistent with the recent growth of the lake surface area from during our study period. Even though we downloaded images with less than 5% cloud cover, during image processing, we noticed some images were still contaminated by clouds. As such, they were disregarded for our analysis. This explains the gaps in Figure 10a. Spatially, water maps show a permanent water body in the southern part of the lake with patches of water appearing in the northern section of the lake depending on the season (Figure 9a). 

We found that flooding events are limited to once a year during our 13-year study period. This is often during or after the rainy season when we have patches of water forming in the northern section. These water patches were considered as water features in this study. During the dry season, most of the water bodies in the northern section of the lake dries up. We recorded no direct link in terms of water out flow from the southern pool into the northern pool of the lake. Hence, the northern side of the lake still solely relies on direct rainfall or an inflow from the Yobe River. The lake bathymetry enhances water loss in this area. With water not being able to flow from the south to the north, this increases chances of evapotranspiration and seepage of the water pool in the southern part [38]. Even though there was a slight increase in lake area during our study period, the large seasonal fluctuations reported to have occurred in the 80s [47], still happens in recent times (Figure 9a).

### 6.3. Error Analysis

An uncertainty encountered during this study was cloud cover. It reduces our data set and makes distinguishing water features difficult. Images with more than 10% cloud cover were disregarded for this study. Only water extraction derived from MNDWI was adopted for error analysis. Errors in extracting our area estimates could be from sensor properties, the lake water characteristics, and data processing. For this study, Landsat 7 ETM+ and OLI 8 images were used for water area extraction.

For the ETM+ image, a high-resolution Google Earth aerial image was used to select a reference image. This was mostly done by careful visual interpretation. This was done by comparing their water boundaries. For the OLI image, a very high-resolution multispectral image from Worldview-3 obtained from DigitalGlobe served as our reference image. Coupled with proper processing techniques, we reduced our error sources to the lakes characteristics.

The water extraction results were all acquired using the Otsu threshold. As Table 5 and Table 6 show, the extraction process was not flawless. Some omission errors were recorded. Figure 5, Figure 6 and Figure 8 show MNDWI properly delineated water from non-water features. However, in the recognition of patches of turbid water around the lake, some omission errors are recorded. This is because, the reflectance of shallow water is usually affected by what is underneath hence affecting its spectral features. This is probably why those areas were missed during the extraction process. Even after we applied a manual threshold with reference to the Otsu threshold, we still observed some omissive errors (Figure 8). Omissive errors around the edges were also identified. Edge pixels are likely to consist of water and non-water features [96]. This can be addressed using spectral unmixing [20,97].

## 7. Conclusions

In this study, we estimated changes in Lake Chad’s area from Landsat 7 and 8 satellite images. MNDWI performed better than other indices for mapping the water extent from acquired Landsat images. When used with the “proper” threshold value, MNDWI can identify open water, turbid water and small water bodies which all makes part of the larger Lake Chad. This study results demonstrated that using a conventional threshold of value 0 could results in high commission errors in this area. However, when provided with reference data, optimized threshold values could be generated for large data set in a reproducible manner using the Otsu algorithm. This helped in producing high accuracy lake area extraction results in this area. Due to the difficulty in delineating some turbid water in our area, when applying MNDWI for future studies in this area, a thorough investigation of the quality of image is required. Estimated lake area generally increased from 2003–2016. From 2011–2016, the lake’s area increased by a further ~314 km^2^ mainly due to increasing wet conditions and less irrigation in the area. GRACE TWSA was used as a representative metric to help explain the lake area changes both seasonally and inter-annually. It could be used as a hydrological indicator for future designs of the Lake Chad’s management strategies. With the constant improvement of the Landsat and GRACE missions, the problem of limited availability of healthy data sets could be addressed by proper use of the free data set in this area.

The FAO proposed a response strategy to the water crisis in this area for the period 2017–2019. A key point they noted towards achieving this goal is to provide mapping an analysis of existing resourceful features around the Lake Chad basin and a generational analysis of land use and water utilization at cross-border levels [53]. Water coverage durations estimated from freely available Landsat 8 imageries could be a step towards achieving this goal. Given the advancements in the OLI sensor, a thorough study using Landsat 8 images will help determine with much accuracy the water coverage seasonal duration as well as other surface features around the lake in 2017–2019.

## Figures and Tables

**Table 1:** Satellite-derived indices used for water features extraction.

Index	Equation	Reference
Automated Water Extraction Index (AWEI)	AWEI=4×(Green−SWIR1)−(0.25×NIR+2.75×SWIR2)	[27]
Modified Normalized Difference Water Index (MNDWI)	$\mathrm{NDWI}=\frac{\mathrm{Green}-\mathrm{SWIR}1}{\mathrm{Green}+\mathrm{SWIR}1}$	[33]
Normalized Difference Vegetation Index (NDVI)	$\mathrm{NDVI}=\frac{\mathrm{NIR}-\mathrm{Red}}{\mathrm{NIR}+\mathrm{Red}}$	[80]
Normalized Difference Water Index (NDWI)	$\mathrm{NDWI}=\frac{\mathrm{Green}-\mathrm{NIR}}{\mathrm{Green}+\mathrm{NIR}}$	[54]

**Table 2:** Description of Landsat 7/OLI scenes and corresponding reference data.

Test Site	Path/Row	Selected Landsat Data		Reference Data	
		Sensor	Date	Source	Date
Lake Chad	185/51	ETM+	15 February 2007	ETM+	8 February 2007
				Google Earth	1 February 2007
		OLI	30 December 2015	Worldview-3 *	22 December 2015

* The Worldview-3 data commercially available at www.digitalglobe.com.

**Table 3:** The principle of the error matrix.

		Predicted	
		Negative	Positive
Actual	Negative	a	b
	Positive	c	d

**Table 4:** Estimated area from each index and their respective threshold for the test years.

	2007		2015	
Reference Area	1350 km^2^		<2000 km^2^	
Index	Land-Water Threshold	Lake Area(km^2^)	Land-Water Threshold	Lake Area(km^2^)
AWEI	−0.2	1012	−0.13	1690
MNDWI	0.07	1394	0.2	2085
NDVI	0.09	1487	0.2	2175
NDWI	−0.18	1549	0.12	2137

**Table 5:** Accuracy assessment analyses.

Index	2007			2015		
	AE (km^2^)	OA (%)	κ	AE (km^2^)	OA (%)	κ
AWEI	429	86	0.82	621	89	0.85
MNDWI	−44	95.7	0.9	−85	97	0.91
NDVI	−137	90	0.85	−175	92	0.89
NDWI	−194	93.2	0.88	−112	95	0.9

**Table 6:** Accuracy comparison of the different threshold of the lake area extracted by MNDWI.

	12 December 2015		
	Threshold	OA (%)	κ
	0	80	0.82
MNDWI	0.2	94	0.91
	0.41	95.8	0.92
	0.52	98.6	0.96
